# The polymer and materials science of the bacterial fimbriae Caf1

**DOI:** 10.1039/d3bm01075a

**Published:** 2023-09-26

**Authors:** David A. Fulton, Gema Dura, Daniel T. Peters

**Affiliations:** a Chemistry-School of Natural Science and Environmental Sciences, Newcastle University Newcastle-upon-Tyne NE1 7RU UK david.fulton@ncl.ac.uk; b Departamento de Química Inorgánica Orgánica y Bioquímica Universidad de Castilla-La Mancha Facultad de Ciencias y Tecnologías Químicas-IRICAAvda C. J. Cela 10 Ciudad Real 13071 Spain; c Biosciences Institute, Medical School, Newcastle University Newcastle upon Tyne NE1 7RU UK

## Abstract

Fimbriae are long filamentous polymeric protein structures located upon the surface of bacteria. Often implicated in pathogenicity, the biosynthesis and function of fimbriae has been a productive topic of study for many decades. Evolutionary pressures have ensured that fimbriae possess unique structural and mechanical properties which are advantageous to bacteria. These properties are also difficult to engineer with well-known synthetic and natural fibres, and this has raised an intriguing question: can we exploit the unique properties of bacterial fimbriae in useful ways? Initial work has set out to explore this question by using Capsular antigen fragment 1 (Caf1), a fimbriae expressed naturally by *Yersina pestis*. These fibres have evolved to ‘shield’ the bacterium from the immune system of an infected host, and thus are rather bioinert in nature. Caf1 is, however, very amenable to structural mutagenesis which allows the incorporation of useful bioactive functions and the modulation of the fibre's mechanical properties. Its high-yielding recombinant synthesis also ensures plentiful quantities of polymer are available to drive development. These advantageous features make Caf1 an archetype for the development of new polymers and materials based upon bacterial fimbriae. Here, we cover recent advances in this new field, and look to future possibilities of this promising biopolymer.

## Abstract 2

Progress in revealing the rich polymer and materials science of Capsular antigen fragment 1 (Caf1), a bacterial fimbriae expressed by the bacterium *Yersina pestis*, is described.

## Graphical abstract

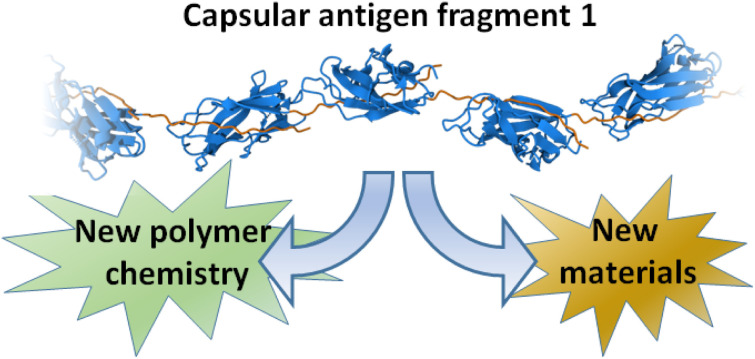

## 1. Introduction

Many Gram-negative bacteria grow hair-like fibres, often referred to as pili or fimbriae, upon their surfaces.^1^ These fibres, which are typically several nanometers thick and up to several microns in length, are polymeric structures composed of chains of non-covalently linked 15–25 kDa protein monomers.^2^ The fibres serve a number of purposes. Some help bacteria adhere to surfaces, serving as anchor chains that allow them to remain attached to tissues.^3^ Some serve as ‘invisibility cloaks’ helping bacteria evade detection from the immune system.^4^ Other functions are thought to include phage binding, DNA transfer, biofilm formation, cell aggregation, host cell invasion and twitching motility (a method by which some bacteria can move across surfaces).^5^

Since the discovery^6^ of these fibres in the 1940s, there has been much work to better understand their structures, assembly, post-translational modifications, regulation of expression and most importantly, their roles in disease. Very recently, however, new questions are beginning to emerge about the fibres produced by bacteria. Can we make materials from these fibres? What uses might there be for these materials? Not so long ago, such questions would be very difficult to answer. Advances in synthetic biology, however, mean that it is now possible to engineer bacteria and culture them on a sufficiently large scale to produce proteins in good yields,^7^ and with the aid of development work, the economically-viable production of useful quantities of fibres for use in high-value applications is now a real possibility. Furthermore, millions of years of evolutionary pressure have ensured that bacterial fimbriae possess features that allow their bacterial wearers to survive; these features are likely to be very different to those found in well-known synthetic and natural fibres, and thus may lead to materials with novel and unusual properties.

In recent years scientists have started to take the first tentative steps to explore the use of bacterial fimbriae in materials. Early work has focused upon curli pili, proteinaceous coiled fibres found on enteric bacteria such as *E. coli* and *Salmonella* spp.^8^ Curli are functional amyloid fibres assembled as part of an extracellular matrix that encapsulates the bacteria within a biofilm, and their application has been witnessed within the emerging field of engineered living materials (ELMs), where living organisms create highly functional materials.^9,10^ Interestingly, curli—which have β-sheet rich structures—share some features with amyloid fibers found in neurodegenerative diseases, such as Alzheimer's and Parkinson's disease, and in prion diseases.^11^ Unlike amyloid fibres involved in disease, Curli are the product of a highly regulated process, and other functional amyloids have been observed in fungi and yeast,^12^ indicating amyloid fibres are not simply the unwanted result of misfolding process, but have evolved in some organisms to serve a purpose. The focus of this review, however, will be upon Capsular antigen fragment 1 (Caf1), the fibre expressed naturally by the well-known plague bacterium *Yersinia pestis.*^13^ Caf1 is very different to curli in its structure and function, belonging to a class of fimbriae which are assembled by the so-called chaperone-usher (CU) pathway,^14^ where chaperone and usher proteins are used to help assemble the fibre and secrete it upon the bacterium surface. Fimbriae assembled by the CU pathway constitute the largest membership,^15^ and although many of these are only known at the genetic level, some such as Type 1 fimbriae,^16^ P pili fimbriae^17^ (both expressed by *E. coli*) and Caf1^13^ have well-characterized structures and their biosynthesis is well-understood.

Interestingly, the membership of the CU family of fimbriae can be divided into six clades (a clade is simply a group descended from a common ancestor) based on genetic similarity: α, β, γ, κ, π and σ, where the γ clade can be subdivided into a further four clades.^15^ Each CU operon (an operon is a group of genes under the control of a single promoter) contains a chaperone, an usher and at least one subunit, with major differences between clades including operon organisation, number of subunit types within the fimbriae and surface structure type. Caf1 belongs to the γ3 subclade, alongside other fimbriae such as the Saf proteins (from *Salmonella enterica*) and the PsaC proteins (also from *Yersinia pestis*). In contrast to Type 1 and P pili fimbriae (which belong to the γ1 and π clades, respectively), Caf1, as well as some other members of the γ3 clade, form “nonfimbrial” surface structures, *i.e.* they do not form rigid rods. Depending on their intended purpose, some CU fimbriae are constructed from multiple types of subunits. For example, Type 1 fimbriae act as anchor chains to tether *E. coli* to tissue surfaces, and thus in addition to many repeats of a subunit which constitutes the length of the linear rod, there are also other types of subunits at the terminus, including an adhesion protein which binds to ligands expressed on cell surfaces. Such proteins can be described as pro-adhesive. Caf1 fimbriae, on the other hand, serves an anti-adhesive purpose (discussed in the following section) and is thus constructed from only a single subunit protein.

This review will explore the structure and function of native Caf1, how it may be engineered to provide defined, bioactive polymers and hydrogel materials, and some early example applications where these engineered materials are being put to use.

## 2. Caf1 function and structure

### 2.1 The function of Caf1

The native source of Caf1 is *Yersinia pestis*, the bacterium infamously responsible for bubonic plague. The bacteria are usually transmitted to a host through a flea bite, where it migrates to the lymph nodes and multiplies, eventually infecting the bloodstream, causing bacteraemia and eventually death. Expression of Caf1 fimbriae is temperature sensitive: it is only produced by the bacteria after it is injected from a (relatively cool) flea into a (warm-blooded) host animal. Presumably on account of the relatively high density of Caf1 fimbriae and their relatively long lengths, they form a gelatinous capsule around the bacterial surface^4,18–20^ (this function is reflected in the name Capsular antigen fraction 1). Thus, Caf1 fimbriae act as a “cloaking device” that shield the bacterium from engulfment and destruction by macrophages (a type of white blood cell whose purpose is to recognize and then ingest foreign cells which are deemed to pose a threat to the host organism) during an infection.^4,21^ More detail on how *Yersina pestis* evade detection is provided in section 3.7.

Caf1's prophylactic ability stems from factors that prevent the recognition of the bacteria by macrophages.^21^ The expression of many Caf1 chains—which display multiple charged residues—upon the bacterial surface provides a brush-like surface which presents a physical barrier to cell interaction. Caf1 fimbriae do not feature any attachment sites onto which macrophage cell surface receptors can bind with high affinity. Caf1 fimbriae also have a surprisingly high mechanical stability, preventing them from breaking to expose the bacterial surface (which macrophages would recognise easily). The combination of these properties makes the Caf1 coat essentially a “non-stick” surface that prevents recognition^4^ (and subsequent engulfment and destruction) of the bacterial by macrophages, and it is hence a major contributor to the pathogenicity of *Yersina pestis* bacteria. Bubonic plague is still a very dangerous disease, and this fact, together with the emergence of drug resistant strains^22^ and the possibility of its use^23^ in biological weapons, has driven research to better understand the biogenesis, structure and function of Caf1.

### 2.2 Caf1 biogenesis

**Fig. 1:**
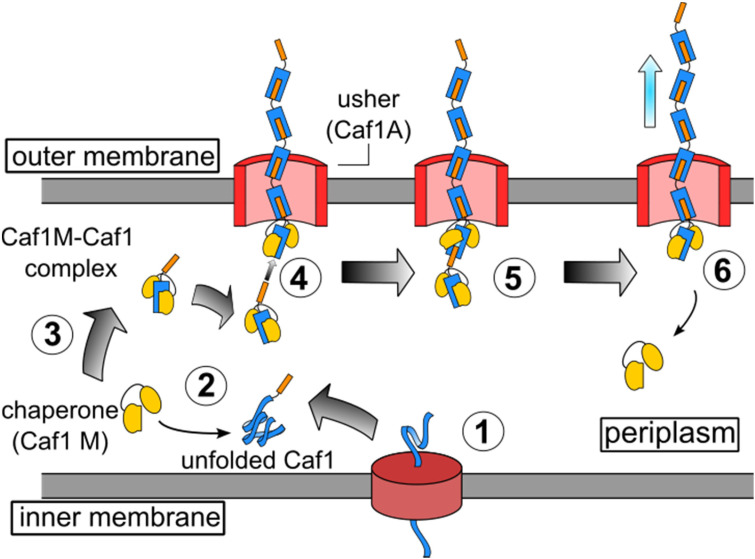
The *in vivo* synthesis of Caf1 by the chaperone-usher pathway.^13^ Step 1. The Caf1 protein (in blue) is secreted in its unfolded form into the periplasm. Step 2. The Caf1 protein folds upon its complexation with a Caf1M chaperone (in yellow) to afford (Step 3) a stable Caf1M : Caf1 complex, which is delivered to the usher protein. Steps 4 & 5. The donor strand of this complex displaces the chaperone from the Caf1 subunit located at the terminus of the growing Caf1 oligomer. This process, termed donor strand exchange (DSE), is mediated by the pore-shaped usher protein (Caf1A). Step 6. The growing Caf1 oligomer is now one subunit longer, and the displaced Caf1M becomes available to complex with another unfolded Caf1 subunit.

Elegant work by Knight and co-workers has led to the elucidation of the mechanism by which Caf1 fimbriae are synthesized,^13^ and being well documented, this aspect of Caf1 will thus be discussed here only in brief. Caf1 fimbriae are constructed from monomeric subunits in a process which is aided by chaperone and usher proteins. These are encoded within an operon consisting of four genes: the regulator (*caf1R*), chaperone (*caf1M*), usher (*caf1A*) and subunit (*caf1*). The Caf1R protein is a non-canonical AraC family transcription factor that is responsible^24^ for the temperature sensitive expression of the *caf1i* gene. Following their translation (Fig. 1), the unfolded monomeric Caf1 subunits proteins are trafficked to the periplasm through the SecYEG pathway, where they are complexed by the Caf1M chaperone, forming a Caf1M–Caf1 complex. This complex delivers the Caf1 subunits to the usher (Caf1A), a doughnut-shaped protein which is located in the outer membrane of the bacteria. Through a process known as donor strand exchange, Caf1A then simultaneously assembles the subunits into a polymer and secretes them to the exterior of the cell, where they form the gel-like protective coat.^25,26^

### 2.3 The structure of Caf1

**Fig. 2:**
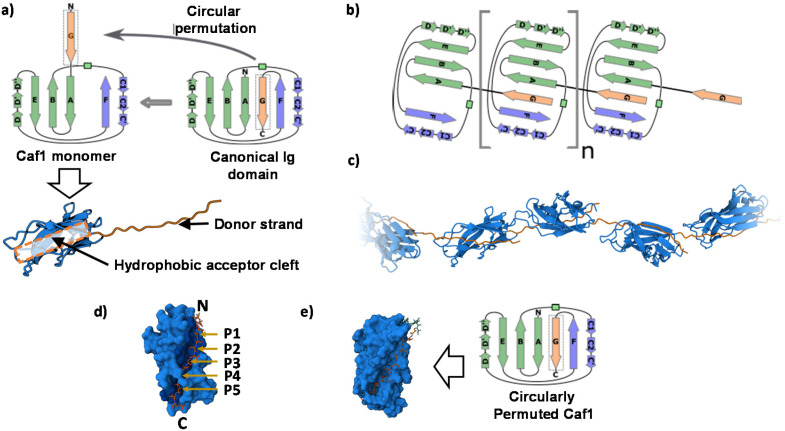
(a) The Caf1 monomer is an immunoglobulin (Ig) domain consisting of a 2-layer sandwich of β-sheets. The strands of one β-sheet are coloured green and the other β-sheet strands are coloured light blue. In a canonical Ig domain, the G-strand (coloured orange) sits at the C-terminus of the polypeptide sequence. In the Caf1 monomer, however, this G-strand—which is usually called the N-terminal donor strand—has undergone a circular permutation that positions it at the N-terminus of the polypeptide sequence. This exposes a deep hydrophobic cleft. As an intersubunit linkage is formed by the complexation of a donor strand by a hydrophobic acceptor cleft in an adjacent subunit, it is useful to think of the monomeric Caf1 subunit as two distinct moieties: a donor strand and a hydrophobic acceptor cleft. (b) Polymerization of Caf1 monomers sees the donation of the N-terminal donor strand into the vacant hydrophobic cleft of the adjacent monomer in the chain, completing its Ig-fold. *n* is simply the average number of subunits in each polymer chain. (c) Model of the Caf1 polymer structure (generated from PDB entry 1P5U). The N-terminal donor strands (colored orange) are complexed by the acceptor domains (colored blue) of adjacent subunits. (d) The Caf1 acceptor cleft complexed by N-terminal donor strand (for clarity, only a single donor–acceptor complex is shown); the five binding pockets (P1–P5) are highlighted. (e) Structure of circularly permuted Caf1 monomer.^38,39^ In this protein the N-terminal donor strand (G) has been located upon the C-terminus, thus allowing the donor to sit within the acceptor cleft in exactly the same way as it would in the polymeric species. This allows the study of the donor acceptor interaction within a monomeric platform which is easier to study than polymeric counterparts.

To date, no X-ray crystal structure (or for that matter, a cryo-TEM) of Caf1 in its oligomeric/polymeric states has been reported. However, key knowledge of the structure of the Caf1 subunit and the nature of Caf1–Caf1 interactions (which link together the subunits) has been derived^13^ by Knight, MacIntyre and co-workers from a 2.2 Å resolution crystal structure of an isolated ternary complex. This complex can be considered as a ‘snapshot’ of a Caf1 subunit during the *in vivo* polymerization process, and the middle subunit of this complex consists of a Caf1 monomer whose structure is thought to be identical to that of a Caf1 subunit situated within an actual polymer chain. The Caf1 monomer (Fig. 2a) is an immunoglobulin domain,^27^ consisting of a 2-layer sandwich of β-sheets around a hydrophobic core. In comparison to a canonical Ig domain, the Caf1 subunit has an incomplete nature; what would be its C-terminal β-strand has been displaced to the N-terminus, exposing a deep hydrophobic acceptor cleft. This particular β-strand, usually called the N-terminal donor strand, is physically constrained from binding into the vacant acceptor cleft in an intramolecular sense. Instead, the N-terminal donor strand binds into the hydrophobic acceptor cleft in an intramolecular sense, completing the immunoglobulin fold of the adjacent subunit (Fig. 2b). This design feature is a remarkably powerful structural trick, transforming an inherently monomeric protein into one with a strong predisposition to form polymer chains. Interestingly, this same design trick has been used^28^ by Jung and co-workers with superfolding green fluorescent protein, which has a β-barrel-like structure where an 11-strand β-sheet forms a closed toroidal structure. By expressing the 11^th^ beta sheet strand onto the N-terminus through a small peptide linker designed to avoid intramolecular association, the protein was engineered into a polymerizable monomer able to form a range of well-defined oligomeric assemblies.

Negative stain TEM studies performed at Newcastle of isolated recombinant wild-type Caf1 polymer (*native*-Caf1^WT^) have revealed^29^ its linear polymeric quaternary structure. Images show a linear beaded structure with turns and bends suggesting a significant degree of chain flexibility, with polymer lengths up to 2 μm observed. Each bead corresponds to an individual protein monomer subunit measuring approximately 2.5 ± 0.4 nm in width and 5.8 ± 1.0 nm in length, values which correspond well with that of a monomer unit (within the isolated ternary complex) as observed by X-ray crystallography. This proof of Caf1's polymeric quaternary structure, together with the X-ray crystal structure showing the monomeric structure, allowed Lakey and co-workers to propose a model of the Caf1 polymer (Fig. 2c). This work was the first time that Caf1 polymers had been visualized, a considerable achievement given the thin nature of the Caf1 polymer makes it challenging to obtain images by TEM. Some other bacterial fimbriae have thicker diameters as their protein chains are coiled into helices which makes them appear as rigid rods, and this feature makes them easier to image (for example, Type 1 fimbriae is 7 nm in diameter, being first imaged in 1949).^30^

### 2.4 The nature of the intersubunit linkage

The donation of a donor strand into the vacant acceptor cleft of an adjacent subunit results in an incredibly strong complex stabilized by a combination of extensive hydrogen bonding network between the strands of the β-sheets and, very importantly, hydrophobic interactions which maintain the protein core.^13^ Work by Zavialov, Knight and co-workers estimates^25^ the free energy of the inter-subunit interaction to be in the region of 70–80 kJ mol^−1^ at 37.5 °C, suggesting the interaction is one of the tightest known protein–protein interactions.^31^ Studies of other chaperone-usher systems have revealed^32,33^ that the intersubunit linkages possess incredible kinetic stabilities, with dissociation half-lives of the order of hundreds of millions of years, and it is speculated^32^ that this stability is a feature of all chaperone-usher systems including Caf1. The exceptionally high kinetic and thermodyanamic stability of the Caf1–Caf1 interaction makes it very different to other protein–protein interactions, which are generally weaker^34^ (typically *K*_a_ ∼ 10^3^–10^9^ M^−1^) and more dynamic^35^ in nature. The high kinetic and thermodynamic stability of the intersubunit linkage contribute to Caf1's high thermostability, even in harsh conditions such as extremes of pH, high salt concentrations and the presence of detergents.^36^ The strength and stability of Caf1 can also be a distinct advantage in applications: the monomers are essentially fixed within the polymer, and thus any materials formed from the polymer will also be very robust. As its protein subunits are linked through non-covalent bonds, Caf1 can be considered as a supramolecular polymer.^37^ However, the high stability of the intersubunit linkage makes Caf1 conceptually more similar to a covalent polymer, where monomer subunits are linked through strong kinetically-fixed covalent bonds. As we outline in section 3.4, however, it is possible to reversibly break and reform the Caf1 intersubunit linkages, thus endowing them with some of the virtues of supramolecular polymers.

Work by Zavialov, MacIntyre and co-workers has shown^38^ how the stability of the Caf1–Caf1 interaction can be weakened through structural mutagenesis of the N-terminal donor strand. In this study, an extensive selection of mutations to the N-terminal donor strand were performed and their effects upon the stability of the folded acceptor–donor complex were determined. The acceptor cleft of Caf1 has five binding pockets (P1–P5) which are occupied by key amino acid residues of the N-terminal donor strand (Fig. 2d). By systematically substituting one or more of these key residues with larger and more hydrophobic residues, it was possible to measure the extent to which the folded acceptor–donor complex was destabilized. These experiments were performed with a circularly permuted^39^ mutant (Fig. 2e), where the N-terminal donor strand has been located upon the C-terminus, thus allowing the donor to sit within the acceptor cleft in exactly the same way as it would in the polymeric species. This monomeric platform makes the study of the donor–acceptor interaction easier than with the polymeric platform, especially as X-ray crystal structures can be obtained. It was observed within the wild-type species that the donor strand sits within the acceptor cleft in a way which allows the two sheets of the β-sheet sandwich to be optimally packed against each other. However, as larger and more hydrophobic residues are substituted into the N-terminal donor stand, the two sheets of the β-sheet sandwich loose their optimal packing. Increasing its sterics thus make the donor strand act as a wedge within the β-sheet sandwich, peeling apart its layers and destabilizing the entire protein. This destabilizing effect is quite small when a single amino acid is substituted, but becomes increasingly larger when multiple substitutions are made (lowering *T*_m_ to ∼25 °C). As will be seen in section 3.7, structural mutagenesis of the N-terminal donor strand also affects significantly the mechanical properties of the Caf1 polymer, with significant biological impact.

## 3. Synthesis, structural modification and applications of Caf1

### 3.1 The recombinant synthesis of Caf1

A significant challenge in the study and development of any recombinant protein is to ensure sufficient quantities are available for experimental work. Originally, methods for producing Caf1 recombinantly—either in its polymeric or circularly permuted forms—were developed for the study and development of Caf1 as an anti-plague vaccine. As the potential of Caf1 as a biomaterial has grown, these synthetic methods have been improved, and it is now possible to regularly produce Caf1 from recombinant *E. coli* cultures in yields of up to 1 g L^−1^.^40,41^ The high yields obtained allow the ready supply of high-purity Caf1 polymer and its mutants required in the development of Caf1-based materials.

### 3.2 Structural mutagenesis of the Caf1 subunit

A significant advantage of Caf1 is that it presents excellent scope for structural mutagenesis, allowing the incorporation of peptide sequences which enhance greatly its potential utility. Some aspects of this were already discussed in section 2.3, where the effects of amino acid substitutions within the N-terminal donor strand upon the stability of the Caf1–Caf1 interaction were described. The focus here is upon the utilization of structural mutagenesis with the aim of incorporating bioactivity or some other useful function into the Caf1 polymer.

**Fig. 3:**
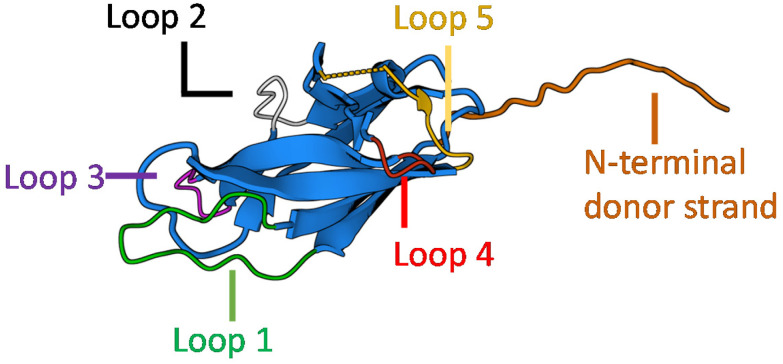
The Caf1 monomer presents a selection of locations (loops 1–5, or at the N-terminus of the donor strand) within the peptide sequence to perform mutagenesis, which can be used to insert biologically active peptide motifs or other functional amino acid sequences.

Caf1's non-stick behaviour towards mammalian cells makes it biologically rather inert. Bioactivity can easily be installed, however, by engineering biologically relevant peptide motifs into its structure. The Caf1 subunit possesses several surface loops (Fig. 3) which potentially allow the incorporated motif to be displayed in a manner which makes it accessible *e.g.* to cell surface receptors. Loop 5 has been found to be particularly accepting of mutations without significant negative impacts on the yield of recombinant synthesis. Initial work^40^ at Newcastle focused on the integrin binding motif, RGDS (Arg-Gly-Asp-Ser), a widely used motif which provides an anchor point for cell-surface integrins.^42^ Longer peptide motifs known to be important in cell culture, including Laminin and VEGF, have also been successfully incorporated within loop 5.^43^ Advantageously, Caf1 mutants containing insertions at loop 5 also appear to possess near-identical physical and spectroscopic properties to *native*-Caf1^WT^, suggesting that insertion at this loop does not alter the protein tertiary/quaternary structure or the stability of the intersubunit linkages.^40^ The addition of peptides to the N-terminus of the Caf1 subunit has also proven to be an effective route to display useful peptide motifs. A useful example^44^ features a hexa-histidine tag (Caf1^His6^), joined to the N-terminus of Caf1 through a flexible linker sequence. His-tags are important peptide motifs which are able to coordinate metals, and are thus often engineered into proteins to aid in their separation and purification.^45^ A single cysteine residue has also been introduced^44^ to the N-terminus of Caf1, which presents an anchor point for conjugation with maleimide-containing biomolecules. N-Terminal addition of peptide motifs is especially useful when longer peptide motifs (≥∼16 residues) are required, which often do not express well within loop 5. Two reported^44^ examples are peptides which mimic the action of osteopontin (OPN) (a 13-mer) and bone morphogenic protein 2 (BMP2) (a 20-mer). These are of interest in bone tissue engineering, where OPN provides adhesion sites to cells through integrin attachment^46^ and can stimulate angiogenesis (the formation of new blood vessels), and BMP2 plays important roles in the differentiation of cells into osteoblasts.^47,48^ These units were expressed within a copolymer, whose biological activity is discussed in section 3.6.

It is important to note that some mutations of Caf1 result in proteins which do not express well *via* the CU pathway (*i.e.* they do not express well in polymeric form); however, it is possible in principle to isolate these species in monomeric form and use an *in vitro* polymerization method to incorporate these subunits into polymer chains (see section 3.4).

Caf1 provides exceptional scope for its structural mutagenesis, allowing its bioactivity to be defined and tuned, useful handles for potential conjugation to be incorporated, and the stability of the inter-subunit linkage to be modulated. In particular, the capacity to ‘hardwire’ peptide motifs into the Caf1 structure is a very powerful feature that allows facile access to bioactive polymers. This feature gives Caf1 a significant advantage over many synthetic polymers, where conjugation chemistries are usually required—which can be often be expensive and/or technically challenging—to decorate polymers with bioactive peptides. There is clearly potential to build up a considerable palette of Caf1 building blocks which will expand greatly the synthetic possibilities of Caf1, ultimately increasing its utility.

### 3.3 The polymer chemistry of Caf1

In polymer chemistry, a linear polymer composed of two or more different monomers is called a copolymer (in some recent Caf1 literature the term ‘mosaic polymer’ is used). Caf1 copolymers present a potentially powerful way to display different peptide motifs upon the polymer chain, or even simply to ‘dilute’ the activity of a bioactive subunit through its copolymerization with a bioinert wild-type Caf1 monomer (Caf1^WT^). These possibilities have driven initial forays to develop methods for the synthesis and characterization of Caf1 copolymers.

**Fig. 4:**
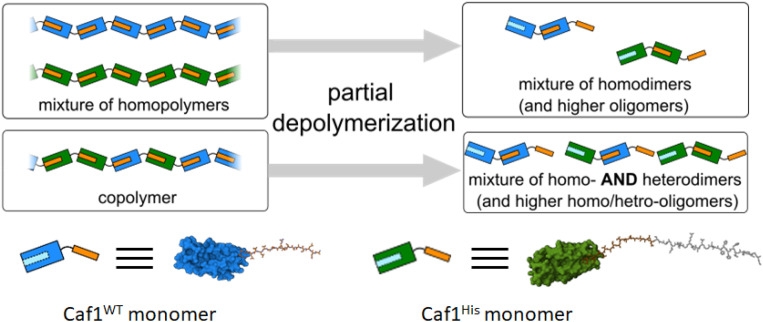
Copolymer or a mixture of homopolymers? The partial depolymerization of a mixture of homopolymers affords only homodimers. The trimer breakdown products (not shown) would simply be two homotrimers (in addition to higher homooligomers, which would also be present). The partial unfolding of a copolymer affords a mixture of homo- and heterodimers (and higher oligomers). The trimer species (not shown) would be an even more complex mixture, containing two homotrimers and two heterotrimers. The presence/absence of homo/heterodimers can be detected by mass spectrometry or gel electrophoresis experiments, and thus the nature of the starting polymer determined.^44,49^

**Fig. 5:**
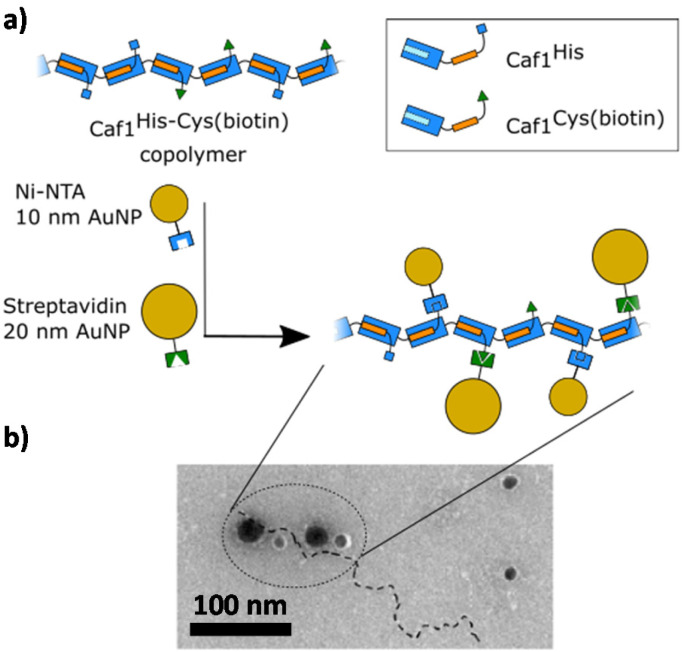
Demonstrating the successful *in vivo* copolymerization of two different Caf1 subunits.^44^ (a) A Caf1 copolymer Caf1^His–Cys(biotin)^, composed of both Caf1^His^ and Caf1^Cys(biotin)^ monomers, was prepared *in vivo*. To confirm the successful copolymerization of both monomers, the copolymer was decorated with Ni-NTA 10 nm Au nanoparticles (which complex with the His tag of the Caf1^His^ subunits) and Streptavidin-coated 20 nm Au nanoparticles (which complex with the biotinylated Caf1 subunits). (b) A TEM image of the Au nanoparticle decorated Caf1 copolymer. The smaller 10 nm and larger 20 nm Au nanoparticles can be observed attached to the Caf1 copolymer chain (highlighted by a dotted line). It is thought that not all His or Cys(biotin) tags are labelled. The observation of both sizes of Au nanoparticles indicates both monomer types have been successfully copolymerized. TEM image adapted with permission from ref. 44. Copyright 2019, BioMed Central.

Work at Newcastle has focussed^44^ on an *in vivo* approach, engineering *E. coli* bacteria to co-express multiple monomers which it was anticipated would then be utilized by the CU system to assemble random copolymers. Briefly, the bacteria were transformed with two *caf1* containing plasmids at the same time, one which is constitutively expressed and a second which is expressed upon addition of arabinose to the culture media. This system can also be expanded by adding multiple *caf1* genes onto the second, arabinose inducible plasmid, to make, for instance, a Caf1 polymer containing three different subunits. In synthetic polymer chemistry, NMR spectroscopic and GPC experiments are usually sufficient to demonstrate that two (or more) monomers have successfully reacted to form a copolymer. Demonstrating that the Caf1 copolymer species is successfully produced and the product is not a mixture of two or more homopolymers is, however, a more challenging task. One approach (Fig. 4) to obtain evidence for the successful synthesis of a Caf1 copolymer exploited the idea that when the Caf1 copolymer is heated close to its melting temperature, subunits unfold with concomitant cleavage of the intersubunit linkage, depolymerizing into dimers and lower oligomers. Assuming that the copolymer has a random arrangement of different subunits, then one would expect the observed dimeric breakdown products to be a mixture of two homo-dimers and a hetero dimer. If, on the other hand, the polymer were simply a mixture of two homopolymers, then the dimeric breakdown products would simply be two homodimers. These hypotheses were tested using a relatively simple SDS-PAGE experiment, with the partial separation of dimer/trimeric bands into their homo- and heterodimeric/trimeric sub-bands observed, thus confirming that a copolymer had been prepared. The success of this experiment requires sufficient mass difference between the homodimer and heterodimer to ensure the successful separation of these ‘sub-bands’ on the gel, and thus one of the subunit types used has to be a significantly different weight to the other. Another method^44^ to confirm the successful synthesis of Caf1 copolymers involved the direct visualization of the copolymer by TEM. This approach utilizes a Caf1 monomer featuring a cysteine residue at its N-terminus, which acts as the anchor point for the conjugation of a biotin tag. This subunit was copolymerized *in vivo* with Caf^His^—which possesses a hexahistdine tag—and the copolymers isolated and then treated with streptavidin-coated 20 nm Au-nanoparticles (which complex to the biotinylated Caf1 subunits) and Ni-NTA nanoparticles (10 nm) (which complex to the hexa-histidine tags of Caf^His^ subunits). TEM images (Fig. 5) reveal Caf1 polymers which have both types of nanoparticle associated with them, confirming their copolymeric nature. This observation, together with the results of SDS-PAGE experiments, support the idea that copolymers have been successfully synthesised *in vivo* by the engineered bacteria.

It is worthwhile highlighting potential limitations and advantages of an *in vivo* approach to copolymer synthesis. At present, it is not possible to control the relative levels of expression of the different subunits by the bacteria (and hence the composition of different copolymers), though use of an alternate expression plasmid could possibly enable this. A potentially powerful advantage of the *in vivo* approach to copolymer synthesis, however, might be in ELMs^9,10^ where the whole organism (together with its secreted polymers and other components) is important. In the following section we describe an *in vitro* approach to copolymer synthesis which potentially allows more control over monomer composition, and also speculate as to some techniques which may more easily allow polymer characterization.

### 3.4 Reversible thermal refolding of Caf1 polymers

Many globular proteins possess a characteristic melting temperature (*T*_m_), the temperature at which the weak non-covalent bonds which maintain structure are broken, causing the protein to unfold with loss of its well-defined tertiary and secondary structures. On account of the highly cooperative nature of these non-covalent interactions, melting transitions are usually very sharp and occur at well-defined temperatures. When cooled, however, many globular proteins do not return to their fully folded states. This irreversibility arises because unfolding usually reveals hydrophobic amino acid residues—which are normally buried in the protein core—that act to drive the irreversible aggregation of protein chains (the classic example of the irreversibility of protein unfolding is the boiling of an egg, which upon cooling does not revert to its initial runny state).

**Fig. 6:**
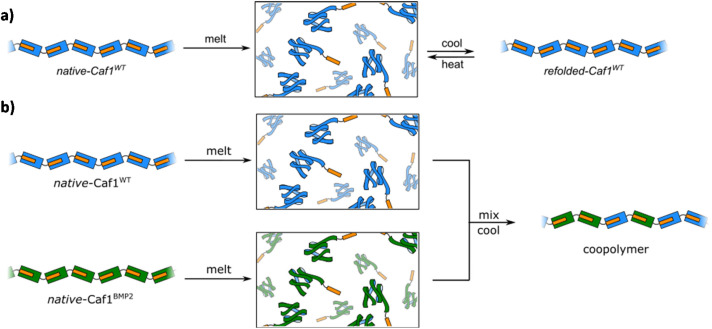
(a) The reversible thermal unfolding of Caf1 polymers. When *native*-Caf1^WT^ is melted, it unfolds with concomitant depolymerisation. When cooled, the monomer subunits refold with concomitant polymerization (the reformed polymer is termed *refolded*-Caf1^WT^, to distinguish it from *native*-Caf1^WT^, which tends to consist of longer chains. *refolded*-Caf1^WT^ can be repeatedly cycled between its polymeric and monomeric forms. b) The *in vitro* synthesis of Caf1 copolymers. Solutions of *native*-Caf1^WT^ (in blue) and *native*-Caf1^BMP2^ (in green) are melted, inducing Caf1 unfolding and depolymerisation. The solutions are mixed and cooled, with the monomers refolding with concomitant polymerization to form the *refolded*-Caf1 copolymer.

When heated at its melting temperature of ∼85 °C (Fig. 6a) the acceptor moiety of Caf1 unfolds, leading to loss of tertiary structure. As the subunit unfolds, the complemented donor strand is released, and thus unfolding also leads to concomitant loss of the polymeric quaternary structure *i.e.* when heated to its melting temperature, the Caf1 polymer depolymerizes. Work at Newcastle discovered^49^ that, quite remarkably, the thermal unfolding of Caf1 is reversible. It was observed that when cooled, the Caf1 protein refolds with concomitant reformation of its polymeric quaternary structure. Thus, temperature can be used to modulate Caf1 between its monomeric and polymeric forms. Key techniques used to support this finding were SDS-PAGE, where the consumption of monomer and the build-up of oligomers was observed as polymerization commenced, and CD spectroscopy, where the kinetics and extent of protein refolding could be ascertained. We speculate that the reversible thermal unfolding of Caf1 is founded upon the capacity of the unfolded subunits—which, unusually, do not appear to aggregate—to rapidly refold to an intermediate form that has sufficient three-dimensional structure to recognize and bind strongly to the N-terminal donor strand of a neighbouring subunit, driving repolymerization.

Experimental observations—including attempts^50^ to follow the progress of polymerization by SAXS—support the idea that the *in vitro* polymerization of Caf1 most likely proceeds by a step-growth polymerization,^51^ where monomers link into shorter oligomers, which then link into longer oligomers and eventually form longer polymer chains. This is in contrast to the *in vivo* polymerization of Caf1 (summarized in Fig. 1), where chaperone and usher proteins are used by the bacterium to assemble polymer chains by adding monomers one at a time onto the end of a growing polymer chain *i.e.* a chain growth mechanism.^51^ In synthetic polymer chemistry it is well established that the key polymer properties of molecular weight (a measure of how long the polymer chains are) and molecular weight distributions (a measure of how broad/narrow are the distribution of chain lengths) depend upon the polymerization mechanism. Thus, an improved understanding of the mechanism of *in vitro* Caf1 polymerization is key in ultimately being able to control polymer lengths and distributions. This, in turn, will allow the design and construction of more complex supramolecular forms of Caf1, which will ultimately increase the number of potential applications for the technology.

An important issue in the development of *in vitro* Caf1 polymerization is the lack of tools to quickly and effectively quantify polymer lengths, distributions and compositions (this issue also applies with characterization of *in vivo* polymers). SDS-PAGE can provide information about distributions of shorter oligomers, but this method is less effective with longer species (>15 mers), and certainly of very limited use with very long chains (>10^2^-mers), where it can be hard to gauge polymer lengths, distributions and modalities with confidence. TEM is a technique with a relatively slow throughput, which in addition to its relative expense, provides limited information with polymers prepared by the *in vitro* method (in principle TEM should provide more information, but the thin nature of Caf1 polymers can make them very difficult to image, especially shorter oligomers). It would be advantageous if there were more techniques available which can afford the determination of molecular weights and distributions. Synthetic polymer chemistry relies heavily upon gel permeation chromatography (GPC) (a type of size exclusion chromatography) for the determination of polymer weights and distributions, however, no well-established chromatographic technique currently exists for polymeric protein species with very high molecular weights (>1 MDa). The development of Caf1 polymer chemistry, and more widely the emerging field of megamolecules with its focus^52–54^ on the synthesis and application of very large and precisely designed protein-based molecules, would certainly benefit considerably from advances in chromatography that allow the separation and quantification of very large molecular weight species. We anticipate improved Caf1 polymer characterization will be most pertinent in biological applications (discussed in section 3.6), where the Caf1 bioactivity will be dictated by the composition (the types of peptide motifs displayed) and polymer length (which dictates the quantity of motifs displayed). It is challenging to determine structure–activity relationships when questions remain around the lengths and distributions of Caf1 polymers, whether they are derived from *in vivo* or *in vitro* synthesis, and so the development of tools which can also more easily quantify Caf1 copolymer lengths, distributions and composition is important.

The meltable feature of Caf1 is significant because it presents new *in vitro* synthetic and materials possibilities. The most obvious is that it offers an *in vitro* approach to prepare copolymers, an alternative to the *in vivo* approach previously discussed in section 3.3. Initial work^49^ used *native*-Caf^WT^ and *native*-Caf1^BMP2^ homopolymers, where the significant mass difference between these two subunits (15 kDa and 17 kDa, respectively) aids in copolymer characterization. In an experimentally simple process, solutions of the two homopolymers were melted, mixed and cooled to room temperature and the copolymer allowed to form (Fig. 6b). Copolymer synthesis was verified by SDS-PAGE experiments of partially unfolded species, and native mass spectrometry experiments—which allowed the masses of lower oligomers to be determined—also supported the successful synthesis of copolymers. An important advantage of the *in vitro* approach to copolymer synthesis is that it offers control over the composition of the different types of Caf1 subunit within the polymer chain. To exploit this feature in cell adhesion, a small library of polymers with systematically increasing quantities of Caf1^RGDS^ (and thus less Caf1^WT^) were prepared. These polymers were then coated onto glass slides and used in cell adhesion studies, which revealed a gradated response *i.e.* the more Caf1^RGDS^ subunits present in the polymer, the greater the cell adhesion. This is a powerful demonstration of the ability to tune polymer bioactivity by modulating the relative composition of the bioactive motif within the copolymer chain. We anticipate that with a wide palette of Caf1 subunits displaying a selection of peptide motifs, together with high-throughput synthesis and screening approaches, it will be possible to rapidly prepare combinatorial libraries featuring a range of compositions and then identify polymer compositions that provide desired bioactivities towards a desired cell type.

### 3.5 Materials from the ‘hair’ of bacteria: Caf1-based hydrogels

**Fig. 7:**
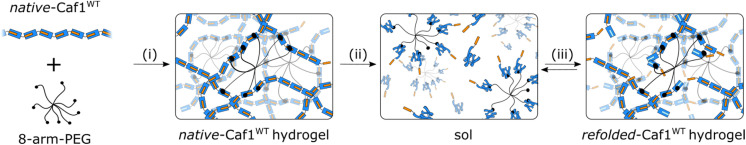
The synthesis and gel to sol transition of Caf1 hydrogels. (i) *Native*-Caf1^WT^ is reacted with a multiarm-PEG whose arms are terminated with *N*-hydroxysuccinamide activated esters (in this example, an 8-arm-PEG is shown) to form *native*-Caf1^WT^ hydrogel. (ii) When the hydrogel is heated at the protein melting temperature (∼85 °C), the Caf1 protein transforms into its unfolded monomeric form, resulting in a gel–sol transition. (iii) When cooled to room temperature, the Caf1 monomers refold with concomitant repolymerization/gelation to form a *refolded*-Caf1^WT^ hydrogel. It is possible to cycle between the *refolded*-Caf1^WT^ hydrogel and the sol.

The desire to further explore the applications of Caf1, especially in cell culture, has motivated the development of Caf1-based hydrogels (the biological applications of the hydrogels are described in section 3.6). Hydrogels are 3D polymer networks possessing high water contents and porous structures, properties they share with the extracellular matrix (ECM).^55–57^ They have recently received great attention on account of their exceptional promise in many applications such as cell culture and tissue engineering.^58,59^ Caf1 polymers in solution do not form hydrogels, even at the upper limits of their solubility in water (∼50 mg mL^−1^),^36^ presumably as there is not sufficient chain–chain interactions or entanglement to cause gelation. Each Caf1 subunit displays seven surface lysine residues, and these can act as anchor points to react with chemical cross-linkers, driving hydrogel formation (Fig. 7, step (i)). Macromolecular PEG cross-linkers possessing ≥2 arms terminated with a NHS-activated ester readily react with the lysine residues of Caf1 to form optically transparent hydrogel materials at relatively low concentrations of Caf1 protein. Low molecular weight cross-linkers, on the other hand, were generally ineffective^40^ at forming hydrogels. After mixing components, gelation times were typically 10–10^2^ s, and the hydrogels formed have high water contents (>95% by weight water), with SEM studies revealing very porous morphologies. As these hydrogels contain similar quantities of both Caf1 (the natural polymer) and PEG (the synthetic polymer), they can be considered as hybrid hydrogels.^60–62^ The mechanical properties of the hydrogels—which can be formulated to have storage moduli in the range 10–10^3^ Pa—were controlled^63^ simply by changing the concentrations of Caf1 used and the relative Caf1 : PEG ratios (typically ∼1–3 mg mL^−1^ protein) and PEG (also ∼1–3 mg mL^−1^ polymer). The storage moduli obtained for Caf1 hydrogels were similar to important hydrogel materials such as Matrigel®, collagen and agarose. There is an increasing awareness^64,65^ that hydrogel mechanical properties are an important factor in the outcomes of cell culture experiments, and thus materials whose rheologies can be tuned are advantageous. Beyond ‘simple’ cross-linking with multiarm PEGs, we envision other cross-linking possibilities with Caf1-hybrid hydrogels. For instance, synthetic polymer crosslinkers featuring disulfide bonds within their chains may introduce potential to trigger hydrogel cleavage *via* addition of a chemical reductant (such as glutathione). This concept might be of use in *e.g.* 3D cell culture applications, where encapsulated cells can be released from the hydrogel matrix through cleavage of the hydrogel network.^66^ There is also the possibility of using alternative types of polymer crosslinker, which may afford hydrogels possessing a wider palette of mechanical properties *e.g.* to make hydrogels with stiffness values several orders of magnitude higher than is easily achievable with PEG.

A basic criterion in the classification of hydrogels is the nature of the cross-linking between polymer chains. In chemical (permanent) networks, polymer chains are crosslinked through strong covalent bonds. In contrast, physical networks feature weak and highly dynamic non-covalent interactions between polymer chains (or between small molecule molecular components, which self-assemble to form long fibres which then entangle). As Caf1 subunits are linked together by non-covalent bonds, there is an argument to consider Caf1–PEG hydrogels as physical hydrogels. However, the exceptionally high kinetic stability of the Caf1–Caf1 intersubunit linkages make them more similar in nature to covalent bonds, and thus it is maybe more appropriate to describe Caf1–PEG materials as chemical hydrogels. The reversible thermal unfolding feature of the Caf1 subunit, however, brings a whole new dimension to Caf1 hydrogels. It was found^49^ that quite remarkably, Caf1's capacity for reversible thermal unfolding was maintained even within the confines of an extensively crosslinked hydrogel network. When the hydrogels were heated at the protein melting temperature (∼85 °C) (Fig. 7, step (ii)), the hydrogel material was observed to transform into a liquid (more formally, it has undergone a gel–sol transition). This observation is consistent with the idea that Caf1 polymers within the network have depolymerized, causing complete breakdown of the crosslinked gel network. When the sample was returned to room temperature it reset back into hydrogel (it has undergone a sol–gel transition) (Fig. 7, step (iii)), indicating that the Caf1 subunits had refolded with concomitant polymerization to reform the hydrogel network. It is quite remarkable that this reversible melting/resetting occurs when one considers that many Caf1 subunits are PEGylated with at least one 20 kDa multiarm PEG cross-linker; presumably this extensive PEGylation does not hinder the capacity of Caf1 subunits to reversibly unfold.

It was found that hydrogels obtained from a cycle of melting/resetting (which are termed *refolded*-Caf hydrogels) possessed different mechanical properties to the starting hydrogels (which are termed *native*-hydrogels because they are formed from Caf1 polymers synthesised by bacteria *via* the CU pathway). *Refolded*-hydrogels were found to possess lower storage moduli than the starting *native*-Caf1 hydrogels, an observation that suggests a change in hydrogel network structure. A possible rationale for this change is that the *refolded*-network has a lower density of cross-links than the original *native*-network. Another factor could be that the *refolded*-network possesses a larger number of dangling chains^67,68^ (terminal chains with a significant length which is not involved in cross-linking). Importantly, when a *refolded*-hydrogel was put through a subsequent cycle of melting/reseting, the hydrogel obtained has identical mechanical properties, suggesting subsequent cycles of melting/resetting see the hydrogel return to the same network structure. Further cycles of melting/resetting also lead to no significant change in properties, suggesting no significant loss in the refolding efficiency of the subunit.

There are many examples of hydrogel materials which possess a thermoresponsive nature, however, the underlying driver of thermoresponsivity in Caf1 materials—the capacity to modulate a globular protein between its folded (and thus polymeric) and unfolded (and thus monomeric) states—is fundamentally different to that operating in other thermoresponsive hydrogels. Thermoresponsive hydrogels based upon synthetic polymers usually exploit the lower critical solution temperature (LCST) phenomenon. This involves polymers which are readily soluble in water at room temperature but which precipitate reversibly from solution when raised above their LCST, changing from an extended chain conformation below this temperature into a collapsed chain above.^69^ This feature allows the polymer to switch from hydrophilic to hydrophobic simply by changing temperature, a switch which can alter dramatically hydrogel properties *e.g.* swelling ratio or pore size. The thermal responsiveness of Caf1 is also fundamentally different to that exhibited by well-known polypeptides such as gelatin and elastin-link peptides (ELPs). Gelatin is comprised of peptides (50–1000 amino acids long) which form a strong triple-helical fibrillar structure. At high temperatures, the polypeptides separate into individual strands, whereas at lower temperatures, some are able to reform their triple-helical structure, forming physical crosslinks that allow the material to gelate.^70,71^ Similarly, ELPs consisting of the repeating (VPGXG)_*n*_ sequence are able to transition from random coil to β-spiral structures above their transition temperature, leading to aggregation and phase separation.^72,73^ The examples presented here highlight that a variety of protein polymers/peptides can display thermoresponsive behaviours, but that it is important to recognize that thermoresponsivity can manifest in different ways and be driven by very different phenomena.

Revisiting the question of whether Caf1 hydrogels are best described as chemical or physical hydrogels, we argue that Caf1 hydrogels have the greatest conceptual similarity to what are termed covalently adaptable networks.^74,75^ These exploit so-called dynamic covalent bonds (DCBs), a term which describes any covalent chemical bond which possesses the capacity to be formed and broken under equilibrium control.^76^ A typical example of a DCB would be an imine bond^77^ (which can be reversibly interconverted to its constituent carbonyl and amine groups), however many other types of functional groups have also been exploited. When DCBs are placed between monomer units in a polymer chain, or to cross link polymer chains into a network, they endow the resultant networks with responsive features, and materials of this class have been extensively studied over the last 20 years or so.^78^ We argue that thinking of Caf1 within the conceptual framework of dynamic covalent materials is a better way to understand the thermoresponsive features of Caf1, differentiating it from other thermoresponsive polymers/materials which may, at first glance, appear superficially similar.

Upon mild deformation, chemically cross-linked networks tend to return to their original shape, and more severe deformation can lead to the irreversible fracture of the material. The meltable feature of Caf1 hydrogels presents them with new material possibilities, and it has been demonstrated^49^ that Caf1 hydrogels can be reshaped, something which is difficult to do with chemically cross-linked hydrogels. The surfaces of Caf1 hydrogels can also be welded together. This process involves heating the faces of hydrogel segments which causes the unfolding of Caf1 subunits situated on or close to the surface of the face. When the faces are joined together, it is presumed that sufficient numbers of Caf1 subunits refold around donor strands of the adjacent face, welding the faces together. This feature may find utility in the preparation of segmented hydrogels, where each segment has different mechanical/physical properties, and thus might be useful in the study of how cells migrate through a material as its properties change between different segments. It was also possible to use the meltable feature to blend two preformed hydrogels together to form a new hydrogel; this feature allows the preparation of hydrogels with a wide range of mechanical properties from a small palette of preformed hydrogels.

### 3.6 Application of Caf1 in cell culture applications

**Fig. 8:**
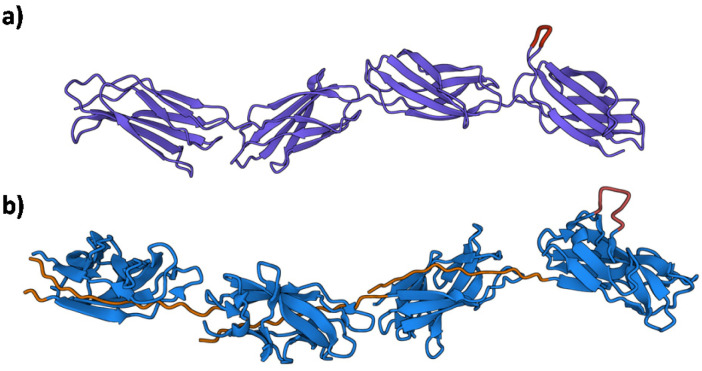
The structural resemblance of the ECM fibronectin with Caf1. (a) Fibronectin Type III domain (PDB File 1FNF) with sites of known cell adhesion motifs (RGDS and the accessory site PHSRN) highlighted in magenta.^91^ Lower molecule in green. (b) Caf1 model based upon X-ray and EM structures.^1,29^

Arguably, the most exciting possibility for Caf1 is its potential in cell culture applications. *In vitro* cell culture—where cells are grown in a lab—is important as there is huge demand for cells in a wide range of biomedical applications including regenerative medicine,^79–81^ disease modelling,^82–84^ personalized therapies^85,86^ and drug testing/discovery.^87,88^ Some cell lines will easily culture, however, many important cell lines such as stem cells, which can differentiate into different types of cells, are more difficult as ensuring the desired outcomes of these experiments can be testing. Key to addressing the challenge is the recreation of complex cell–cell and cell–matrix interactions observed *in vivo*. This has led to an extensive effort both in academia and industry focussed upon the development of artificial biomaterials to mimic the protein network in the extracellular matrix (ECM). Some of the most important artificial ECMs used in cell culture applications are based upon basement membranes, ill-defined gelatinous extracts of proteins and small molecules such as collagen, laminin, and various growth factors derived from the Engelbreth–Holm–Swarm mouse tumours.^89^ These materials—the most arguably well-known of which is Matrigel®—have been used extensively for 3D cell culture on account of their strong record in successfully promoting cell proliferation and differentiation. The disadvantages of these materials, however, remain their high cost, malignant source—which eliminates any potential translation into the clinic—and significant batch-to-batch variation in composition, which can lead to significant issues with reproducibility.^90^ There is thus a significant need in cell culture applications for animal-free materials with well-defined compositions and whose bioactivities can be finely tuned. Caf1, with its structural similarly to the ECM fibronectin^91^ (Fig. 8), demonstrates excellent potential in this regard.

Although Caf1^WT^ polymers are bioinert towards mammalian cells, bioactivity can be engineered easily into subunits (outlined in section 3.2). Table 1 lists the Caf1 mutants used in cell culture studies to date. The first work to establish the potential of Caf1 in cell culture was performed^40^ at Newcastle in 2014. Glass slides were coated with either Caf1^WT^, Caf^RGDS^ or commercially-available collagen IV or fibronectin (two well-known ECM proteins derived from animal sources) and cell viability, adhesion and cell morphology ascertained with 3T3 and PC12 cells. Results revealed the importance of the engineered RGDS motif, with significantly higher levels of cell adhesion with Caf1^RGDS^ than was observed with Caf^WT^. Significant differences were also observed in cell morphology, with cells cultured upon Caf1^RGDS^ and fibronectin displaying cytoplasmic projections called filopodia, whereas those culture on Caf1^WT^ maintained a rounded shape. This change in morphology arises because the cells are recognizing the RGDS motifs, triggering cellular processes that result in a change in their morphologies, and demonstrate that the incorporation of the RGDS peptide motif endows the Caf1 polymer with bioactivity which enables it to mimic fibronectin. Comparisons between Caf1 and collagen are more difficult to make in this study as PC12 cells are known to grow very well upon collagen.^92^ This work provided the initial proof of concept for the use of Caf1 in cell culture applications, as well as suggesting more broadly at the potential biotechnological uses of CU proteins.

**Table 1:** Summary of bioactive Caf1 mutants which have been used in cell culture studies

Caf1 mutant	Nature of the mutation and intended bioactivity	Application in cell culture studies
Caf1^RGDS^	Insertion of RDGS peptide sequence in loop 5: presents binding motif for cell surface integrins	Demonstration that Caf1^RGDS^ polymers or Caf1^WT–RGDS^ copolymers promote the growth and proliferation of human dermal fibroblasts^98^
Caf1^OPN^	Insertion of OPN peptide sequence at N-terminus: provides adhesion sites to cells through integrin attachment	Demonstration that Caf1^OPN–BMP^ copolymers direct the early phases of bone formation^44^
Caf1^BMP2^	Insertion of BMP2 peptide sequence at N-terminus: promotes differentiation of cells into osteoblasts	Demonstration that Caf1^OPN–BMP^ copolymers direct the early phases of bone formation^44^
Caf1^YIGSR^	Insertion of YIGSR peptide sequence in loop 5: promotes cell adhesion and proliferation	Demonstration that synergistic display of Caf1^YIGSR^ and Caf1^VEGF^ can drive angiogenesis of endothelial cells^43^
Caf1^VEGF^	Insertion of VEGF peptide sequence binding motif for cell surface integrins	Demonstration that synergistic display of Caf1^YIGSR^ and Caf1^VEGF^ can drive angiogenesis of endothelial cells^43^

The potential utility of Caf1 copolymers was also explored^44^ in bone tissue engineering. The *in vivo* approach (section 3.3) to copolymer preparation was used to prepare Caf1^OPN^–Caf1^BMP2^ copolymers which were then coated onto plastic, and the surfaces seeded with cells. The motivation to display both OPN and BMP2 motifs upon the Caf1 polymer was inspired by work^93^ which had shown that synergistic display of *both* OPN and BMP2 motifs is required to stimulate the early stages of bone formation by primary human bone marrow stromal cells, a class of cells which are important in bone tissue engineering. This also provided an opportunity to determine whether engineered Caf1 polymers could influence more complex biological responses, in this case differentiation, in addition to simply providing an adhesive substrate for cells. The successful observation of mineralization indicates that the cells were stimulated by the synergistic co-display of the OPN and BMP2 upon the Caf1 polymer, directing the early phases of bone formation. These observations are significant as they demonstrate the potential of Caf1 in tissue engineering applications.

On account of experimental simplicity, the culturing of cells is often performed on flat hard surfaces (such as glass or polystyrene), however, cells cultured in these environments often display aberrant behaviours *e.g.* flattened shape, abnormal polarization, altered response to pharmaceutical reagents, and loss of differentiated phenotype.^94^ Hydrogel materials often present a far superior platform for the culture of cells as they better mimic inherent features of native ECMs.^95^ Like ECM, hydrogels are also mostly water. Hydrogel mechanics are also similar to soft tissues, and can be engineered to provide suitable microenvironments for cell adhesion, proliferation, and migration, and promote the exchange of nutrients and signalling molecules.^55–57^ Extensive research in recent decades has proven^95–97^ hydrogels to be an incredibly useful platform in a range of cell culture applications, allowing for the culture and directed differentiation of various cell types in ways not possible with conventional culture substrates. This raises an important question: are Caf1-based hydrogels useful in cell culture applications? On paper, they certainly have some potentially very appealing features: structural similarly to fibronectin, a capacity to be ‘hardwired’ with important peptide motifs, an ability to be formulated into hydrogels with controlled mechanical properties, and all within an animal-free platform. These features, together with the synthetic and materials properties offered by its meltable nature, suggest Caf1 has considerable potential worthy of further investigation.

Work with Caf1 hydrogels has initially focussed^98^ on the application of Caf1–PEG hydrogels for the culture of human dermal fibroblasts (hDFBs), an appealing cell line for preliminary work as these cells are sensitive to their microenvironment and are easy to interrogate. Results of cell culture studies reinforced the observation that Caf1^WT^ is rather bioinert, and that hydrogels featuring bioactive Caf1^RGDS^ polymers are required to encourage cell proliferation, metabolism and production of collagen. Interestingly, it was observed that when Caf1 copolymers of Caf1^RGDS^–Caf1^WT^ were used, a similar cellular response was observed as when cells were cultured upon hydrogels that used only Caf1^RGDS^. This observation indicates that bioactivity can be maintained even when the bioactive Caf1^RGDS^ subunits are ‘diluted’ with bioinert Caf1^WT^ subunits, a feature that could be very useful when working with some of the more complex versions of Caf1 that are expressed at lower levels and are thus less available. It was also observed that cells grown upon Caf1^RGDS^ hydrogels display more favourable morphologies (polygonal morphologies, larger cytoskeletons) than those grown on stiff tissue culture plastic, emphasising some of the advantages of culturing cells on hydrogels. In this work it was also discovered that cell growth and metabolic activity were higher in a softer *refolded*-Caf1^RGDS^ hydrogel (hydrogel which has gone through a cycle of melting/resetting) in comparison to the stiffer *native*-Caf1^RGDS^ hydrogel (the hydrogel made from the chemical cross-linking of *native*-Caf1^RGDS^). This observation was initially surprising, as cell growth is usually slower on softer materials.^99–101^ It was postulated that the underlying cause of this difference is likely to arise on account of very different mechanical properties of these hydrogels. The stiffer *native*-Caf1^RGDS^ was shown to possess a very elastic nature, and this elasticity likely impact how cells behave on the hydrogel. As cells grow they often remodel their microenvironments by ‘pushing on’ ECM;^65^ as the cells push on an elastic ECM network, the network tends to push back on the cell, making it harder to remodel. The *refolded*-Caf1^RGDS^ hydrogel, on the other hand, was found to be significantly more viscoelastic. Viscoelastic networks tend to yield when pushed, and are thus easier for cells to remodel, and this difference in mechanical properties most likely explains the differences in observed cellular behaviour.

This initial work with Caf1 hydrogels involved culturing cells on top of the hydrogel and is thus very 2D in nature. However, 2D cell culture does not mimic the inherently 3D native *in vivo* cellular microenvironment; cells cultured in 2D lack the cell–cell and cell–matrix interactions that are found in native 3D microenvironments, and this deficiency can contribute to aberrant cell behaviours.^102,103^ Consequently, there has been a significant drive to develop 3D cell culture approaches.^95,102,103^ 3D Cell culture requires that cells be encapsulated within the hydrogel network. Although cell encapsulation is a straightforward task to accomplish with physical hydrogels, it is harder with chemical hydrogels because a covalently cross-linked network has to be formed around the cells. The encapsulation step must also ensure excellent levels of cell viability, and thus the cross-linking chemistry used must be carefully considered. Recent years have seen a wide range of biorthogonal crosslinking chemistries developed for this purpose. These are characterized by their reliance upon unnatural functional groups which react together in high yields under very mild conditions with no by-products such as Diels–Alder,^104,105^ Michael additions,^106^ and strain-promoted azide–alkyne cycloadditions.^107,108^ These biorthogonal chemistries avoid issues associated with toxicity, however, a drawback is that they can introduce extra synthetic complexity and cost into the cell encapsulation process, and some chemical knowledge is usually beneficial when performing these chemistries (especially if there is a need to synthesize the reagents in-house). The meltable feature of Caf1 hydrogels provides a straightforward route to achieve 3D cell encapsulation. In a simple demonstration,^109^ the hydrogel was melted and cooled to room temperature where the gel starts to reform. The gelation time was sufficiently long enough to allow the addition of live cells, and as the hydrogel set these cells became homogenously dispersed within the hydrogel matrix. Work showed that cells could be encapsulated up to 2 × 10^6^ cells per mL (higher densities were not investigated), and that the vast majority of cells survived the cell encapsulation process, indicating its cytocompatibility (presumably when Caf1 is in its unfolded state, the N-terminal donor strand and vacant acceptor cleft do not interact in any significant way with live cells). The presence of RGDS motifs was again found to be crucial in encouraging cell proliferation, metabolism and F-actin production. The RGDS concentration within the Caf1 hydrogel was estimated to be in the 0.05–0.10 mM range, which is very similar to that found in natural ECM.^110^ Results also further supported the idea that the Caf1^WT^ subunit is bioinert and does not cause cellular inhibition or toxicity. Cells were successfully maintained in the hydrogel for 21 d, indicating that Caf1 hydrogels have potential in applications that require longer culture times. This study also benchmarked Caf1 hydrogels against the commercially-available animal-derived basement membrane hydrogel Geltrex®, with the Caf1 hydrogel being observed to perform at least as well as Geltrex® in all assays.

It is often desirable at the conclusion of 3D cell culture to be able to harvest the cells, which requires the hydrogel network to be cleaved to allow cell isolation. This issue remains to be addressed for Caf1 hydrogels, which on account of the cell incompatible temperatures required to melt the hydrogel (∼85 °C), do not offer this feature. One approach could be the use of PEG cross-linkers featuring cleavable disulfide bonds, where GSH might be used to reductively cleave the cross-linkers and the hydrogel network to break down, thus releasing encapsulated cells. Another possibility is to exploit Caf1 mutants where the donor strand has one or more mutations that destabilize the donor acceptor interaction (as discussed in section 2.3), allowing it to be melted at a temperature which allows the matrix to be cleaved but which is not too hot to damage cells. Finally, it may be possible to engineer selectively-cleavable variants of Caf1, which can be specifically cut by protease enzymes at a defined number of points in the Caf1 chain, thus making Caf1 hydrogels biodegradable either by cells or a user.

Within the field of tissue engineering there has been much interest in recent years in angiogenesis, the process whereby new blood vessels are formed.^111,112^ Attempts to promote vascularization of biomaterials have utilized growth factors (these are simply molecules capable of stimulating a variety of cellular processes including cell proliferation, migration, differentiation and multicellular morphogenesis), which are required to stimulate the adhesion and migration of endothelial cells. Often these growth factors are found within basement membrane proteins such as laminin, collagen or fibronectin, whose animal origins may present hurdles to clinical translation. The capacity of Caf1 to provide cells with the required pro-adhesive and pro-angiogenic signals to drive angiogenesis in hydrogel materials has recently been reported^43^ by Simon-Yarza and colleagues. The group have developed a platform hydrogel material based upon the pharmaceutical grade polysaccharides pullulan and cationic dextran, which provides a low-cost bulk material for cell culture. This material has been engineered to possess microchannels, which present a template for the growth of blood vessels. The hydrogel is not itself sufficiently bioactive to promote the desired cellular behaviours required for angiogenesis and so the group have developed a simple method to coat the pores and microchannels of the hydrogel with two Caf1 mutants. One displays the YIGSR peptide motif (*native*-Caf1^YIGSR^), which is known to promote cell adhesion and proliferation, and the other displays the VEGF peptide motif (*native*-Caf1^VEGF^), which is known to promote cell migration. Caf1 possesses a net negative charge and thus readily adsorbs upon the pores and internal surfaces of the hydrogel (which possesses a net positive charge). Work showed that the synergistic display of *both* Caf1^YIGSR^ and Caf1^VEGF^ was required to promote angiogenic cell behaviour, and when displayed individually the desired outcome was not obtained. This proof of concept, with its spatially controlled presentation of Caf1 guiding endothelial cell behaviour, very much highlights the power of Caf1 in advanced cell culture applications as a potential superior alternative to animal-derived basement membrane proteins.

### 3.7 Understanding the role of Caf1 in macrophage engulfment

*Yersina pestis* has a variety of mechanisms to avoid macrophage phagocytosis.^113^ Upon injection from cold-bloodied feas to warm-bloodied mammals, genes responsible for the production these virulence factors are activated. The F1 capsule is expressed, surrounding the bacteria and preventing its association with macrophages.^4^ F1 antigen, once expressed, may render *Yersina pestis* even more able to resist uptake and to rapidly multiply extracellularly, leading to a lethal systemic infection. In addition to its capsule, the F1 antigen, it possesses a secretion system able to inject antiphagocytic proteins into cells.^114–116^ Early in the infection process, there also is a transition period when the bacterium is susceptible to recognition and phagocytosis by macrophages. Upon its phagocytosis, the bacterium inhibits phagosome acidification, preventing its breakdown, and is then able to manipulate host factors that subvert phagosomal maturation and generate a protective replicative niche within the macrophage; in effect, the bacterium is able to multiply within the macrophage.^117^ Bubonic plague continues to be a risk to health, and in addition to driving fundamental interest in the mechanisms of its virulence, this also drives considerable interest in further research to develop new vaccines and treatments.^118–120^

The details of how *Yersina pestis* avoids macrophage phagocytosis are still not fully understood. To gain new insight, work at Newcastle has exploited^21^ the ability to engineer bacteria to express Caf1 chains with structural mutations. *Escherichia coli* bacteria were engineered to express Caf1 (*Escherichia coli* are far easier bacteria and safer to work with than *Yersina pestis*), with TEM images revealing the expression of Caf1 capsules around each bacterium (and which bear a near-identical appearance to the capsules observed around *Yersina pestis*). Fluorescence microscopy showed expression of a Caf1^WT^ coating significantly lowers association between bacteria and macrophages, and even those bacteria which were bound were protected from macrophage engulfment. The underlying reason for poor adherence may be attributable to the net negative charge of Caf1 (its pI is 4.5), which helps repel it from the negatively charged macrophage surface. There is also the possibility that Caf1 is highly hydrated, with a water layer providing a kinetic barrier to binding, a well-known phenomenon observed with synthetic polymers such as PEG, which can also resist protein binding.^121^ The surfaces of macrophages are decorated with integrins, cell surface receptors which can bind to ligands displayed on other species. It was found that *Escherichia coli* expressing Caf1^RGDS^ adhere to macrophages at a very similar level to those expressing Caf1^WT^, however, those bacteria which do adhere were observed to become engulfed. This observation suggests that the binding of RGDS motifs by macrophage surface integrins triggers engulfment. The application of mechanical forces by macrophages upon their targets is also known to be an important role in the processes of recognition and engulfment,^122^ which raises the question as to whether a mechanical feature of the Caf1 polymer may also be a contributing factor in its ability to prevent phagocytosis. It is possible to gauge the mechanical strength of a protein through single molecule force spectroscopy (SMFS).^123^ A protein can be anchored between a fixed surface and an AFM tip, and then a pulling force is applied to the tip which causes the protein to unfold. By measuring the forces encountered during the pulling process, it is possible to estimate the mechanical stability of the protein. Based on these experiments, the unfolding force of Caf1^WT^ was estimated to be 394 ± 40 pN, which is high for a globular protein (which typically unfold at 25–250 pN),^124^ but similar to another well-known CU protein, FimA.^125^ Amino acid substitutions within Caf1's N-terminal donor strand were shown to lower the unfolding force by about 20%. Importantly, lowering of Caf1's stability to pulling forces was found to have a significant impact upon the ability of the Caf1 coating to protect the bacterium from phagocytosis, with the bacteria now being readily engulfed. These mutations do not affect the ability of *Escherichia coli* to produce a Caf1 coating (the coatings produced appear identical to Caf1^WT^), and the donor strand mutations do not influence the ability of the bacteria to be recognized by the macrophage. Rather, it is proposed that the mutation reduces the strength of the Caf1 polymer chains, making them more brittle. Consequently, when mechanical forces are applied by the macrophage, the Caf1 polymer chains break to reveal underlying surface features of the bacteria—so-called pathogen associated molecular patterns—which are recognized by the macrophage, triggering phagocytosis. It is interesting that only a 20% reduction in the mechanical strength of the protein has such a significant reduction in its ability to help the bacteria evade phagocytosis. A likely explanation is the theory of protein marginal stability,^126^ the idea that proteins have evolved ‘just enough’ stability to ensure their function. Thus, a 20% drop in stability may be ‘just enough’ to cancel the anti-phagocytoic function of Caf1. This study has provided important insights to the mechanism by which *Yersina pestis* is able to evade detection by the immune system, and fundamental to this study is the exploitation of the ability of Caf1 to undergo structural modifications.

## 4. Outlook and conclusions

The body of work described in this review represents the tentative first steps to use bacterial fimbriae as a building block for the construction of new materials. It is has also seen Caf1 considered within the framework of polymer chemistry. Building upon decades of extensive work to understand the biogenesis of Caf1 and its role in disease, a new line of investigation is now emerging to develop the supramolecular, polymer and materials science of this bacterial fimbriae.

Caf1, with is structural resemblance to ECM polymers, already appears to have found its application by helping to address a key challenge in the cell therapy and cellular agriculture fields around how to provide biological signals to cells on an industrial scale. Currently, this is achieved through the use of individual growth factors and ECM proteins, often produced recombinantly, but their high prices make the economics of producing cells on this scale challenging. Moreover, they have to be added separately and in the right proportions, are often unstable, and must be replaced during media exchanges. Caf1 presents an entirely new way of supplying bioactivity to cells. The key innovation is that, rather than trying to faithfully reproduce a natural growth factor, Caf1 presents a stable and adaptable scaffold in which can be inserted only the small part of the growth factor necessary for bioactivity. The stability of the Caf1 scaffold makes it easier to work with and ensures longer term activity, and so unlike growth factors, it is likely that it will not have to be added repeatedly. The fact that Caf1 can be immobilized upon surfaces also means that it remains present during media exchanges. The modularity of Caf1 is highly advantageous, as copolymers possessing different peptide motifs can be co-presented for synergy. Furthermore, the number of copies displayed can be defined, allowing the strength of the biological signal to be tuned and optimized for desired outcomes. It is clear from work to date that Caf1 has the potential to be a transformative platform technology in cell culture.

We also anticipate that there is considerable scope to expand beyond Caf1 and investigate the possibilities presented by other CU proteins. For example, Type 1 fimbriae and P pili (both expressed by *Escherichia coli*) display^127^ highly unusual nanomechanical properties similar to a constant force spring, which means that as they are extended, energy is dissipated. This makes them very different to many synthetic polymers, which behave more like Hookean springs *i.e.* as they are stretched they store energy. This feature, together with potential for scalability and molecular-level modifications of their structures, suggest these fimbriae may also make excellent building blocks for materials that are able to relax stress. There are hundreds of other bacterial fimbriae, many of which are only known at the genetic level, and it is possible that many of these have evolved interesting structural and mechanical properties which might be exploited by scientists and engineers. We hope that our review of the rapid progress made developing a polymer and materials science of Caf1 will also inspire further interest in the utilization of Caf1, and also the exploration of other CU systems for potential biotechnological development.

## Conflicts of interest

DTP is a director at MarraBio Limited, an active company incorporated on 10 August 2022 with the registered office located in Newcastle upon Tyne, UK.

## Supplementary Material
